# Synergistic antifungal interactions of amphotericin B with 4-(5-methyl-1,3,4-thiadiazole-2-yl) benzene-1,3-diol

**DOI:** 10.1038/s41598-019-49425-1

**Published:** 2019-09-10

**Authors:** Barbara Chudzik, Katarzyna Bonio, Wojciech Dabrowski, Daniel Pietrzak, Andrzej Niewiadomy, Alina Olender, Katarzyna Malodobry, Mariusz Gagoś

**Affiliations:** 1 Department of Cell Biology, Institute of Biology and Biochemistry, Maria Curie-Skłodowska University, Akademicka 19, 20-033 Lublin, Poland; 2 Department of Anaesthesiology and Intensive Therapy Medical University of Lublin, Jaczewskiego 8, 20-954 Lublin, Poland; 3 Institute of Industrial Organic Chemistry, Annopol 6, 03-236 Warsaw, Poland; 4 Department of Chemistry, University of Life Sciences in Lublin, Akademicka 15, 20-950 Lublin, Poland; 5 Chair and Department of Medical Microbiology, Medical University of Lublin, Chodźki 1, 20-093 Lublin, Poland; 6 Department of Nurse and Health Science, Medical Division in University of Rzeszów, Al. Rejtana 16A, 35-310 Rzeszów, Poland

**Keywords:** Antibiotics, Clinical microbiology, Fungal infection

## Abstract

Amphotericin B (AmB) is a very potent antifungal drug with very rare resistance among clinical isolates. Treatment with the AmB formulations available currently is associated with severe side effects. A promising strategy to minimize the toxicity of AmB is reducing its dose by combination therapy with other antifungals, showing synergistic interactions. Therefore, substances that display synergistic interactions with AmB are still being searched for. Screening tests carried out on several dozen of synthetic 1,3,4-thiadiazole derivatives allowed selection of a compound called 4-(5-methyl-1,3,4-thiadiazole-2-yl) benzene-1,3-diol (abbreviated as C1), which shows strong synergistic interaction with AmB and low toxicity towards human cells. The aim of the present study was to investigate the type of *in vitro* antifungal interactions of the C1 compound with AmB against fungal clinical isolates differing in susceptibility. The results presented in the present paper indicate that the C1 derivative shows strong synergistic interaction with AmB, which allows the use of a dozen to several dozen times lower AmB concentration necessary for 100% inhibition of the growth of pathogenic fungi *in vitro*. Synergistic interactions were noted for all tested strains, including strains with reduced sensitivity to AmB and azole-resistant isolates. These observations give hope for the possibility of application of the AmB - C1 combinatory therapy in the treatment of fungal infections.

## Introduction

Treatment of fungal infections is becoming an increasingly serious medical problem due to the constantly growing number of immunocompromised patients who are treated for autoimmune as well as cancer and allergic diseases and patients after organ or bone marrow transplantations. The arsenal of potential antifungal drugs in comparison with antibacterial agents is very limited. Currently, only several classes of antifungal drugs are used, i.e. polyenes (interacting with ergosterol contained in the cell membrane), azoles (blocking the ergosterol synthesis pathway), echinocandins (inhibiting the synthesis of the cell wall components), and flucytosine derivatives (toxic pyrimidine analogues). The most commonly applied azoles, e.g. fluconazole, itraconazole, voriconazole, and posaconazole, have fungistatic effect, often induce resistance, and exhibit selective activity against fungal pathogens^1,2^. In turn, such drugs as 5-fluorocytosine or flucytosine cause a rapid increase in resistance and should be used in combination with other antifungal agents. Drugs from the group of echinocandins, targeted at the cell wall synthesis in susceptible pathogens from *Candida* and *Aspergillus* genera, are ineffective in fighting many other classes of fungi. The appearance of strains with reduced sensitivity to echinocandins is an increasing problem as well^3^. Among the polyene drugs, amphotericin B (AmB) has been used for the longest time as a first-line of defense in treatment of severe, life-threatening mycoses. Despite the long-term application of AmB, strains with acquired resistance to this antibiotic occur rarely^4^. A widely accepted mechanism of AmB action is its interaction with ergosterol contained in the fungal cell membranes, leading to increased permeability to ions and small organic molecules. The loss of osmotic balance leads to fungal death^5–7^. Systemic treatment with AmB is associated with severe side effects, in particular nephrotoxicity and hepatotoxicity. Given the adverse reactions, the plasma AmB concentration in clinical practice should not exceed 1–2 µg/mL; therefore, it is ineffective against strains for which the MIC value is higher than 1 µg/mL, e.g. for *Candida* strains other than *albicans*, especially *C*. *glabrata* and *C*. *parapsilosis*^8^. To reduce the toxicity of AmB, liposomal, colloidal, and lipid complex forms have been developed, which however do not sufficiently eliminate the side effects^8–10^. The synthesis of different types of AmB derivatives has usually resulted in reduced toxicity, but reduced the antifungal activity at the same time^11,12^. Attempts to obtain AmB complexes with metal ions have also been made. The AmB-Cu(II) complex showed increased solubility in aqueous solutions and reduced cytotoxicity *in vitro*^13,14^. However, the limited stability of the AmB-Cu(II) complex hinders its clinical use.

**Figure 1:**
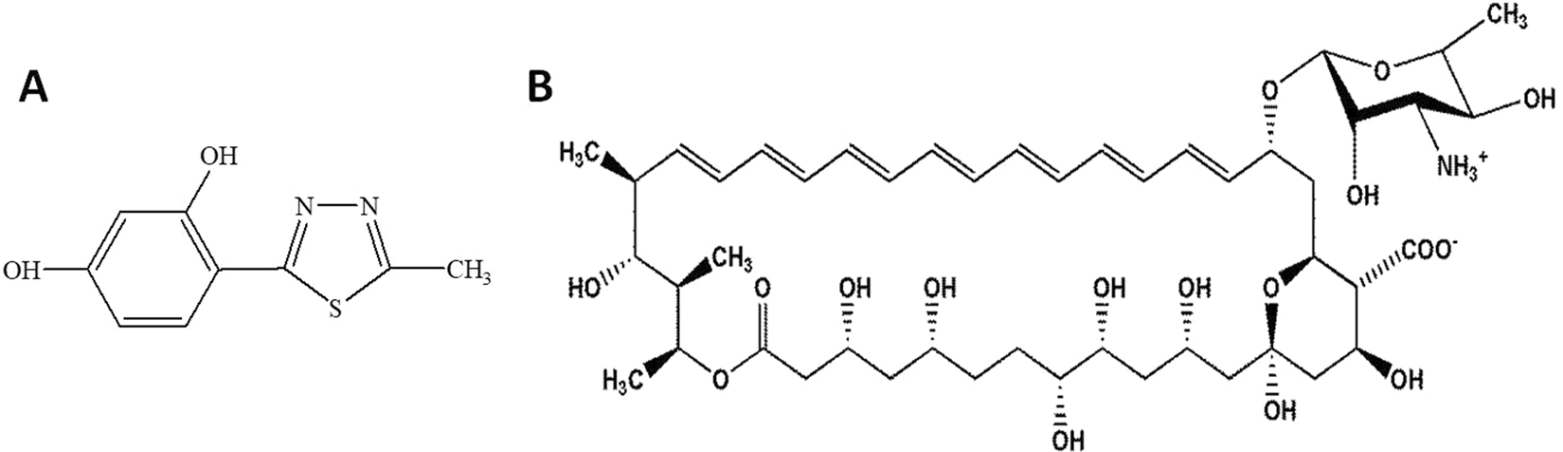
(**A**) Chemical structure of 4-(5-methyl-1,3,4-thiadiazole-2-yl) benzene-1,3-diol (C1); (**B**) chemical structure of AmB.

Despite its high toxicity, AmB is the most effective antifungal with a broad spectrum of activity and rare resistance. A promising strategy to minimize the toxicity of AmB is to reduce its dose by combination therapy with other antifungals, showing synergistic interactions. Therefore, substances that display synergistic interactions with AmB are still being searched for. As shown in many publications, the combination or sequential treatments with AmB and inhibitors of ergosterol biosynthesis (azoles) fail to produce synergistic or additive interactions and often give antagonistic effects, since ergosterol is the target molecule for AmB^15–24^. Although some articles demonstrate beneficial effects of combination therapy with AmB and some azole drugs, it is still regarded as controversial and is usually not applied in clinical practice^25–28^. Enhanced AmB activity was evidenced in combination therapy with cilofungin, i.e. an echinocandin antifungal agent, disrupting cell wall biogenesis^29^. Attempts to apply a combination treatment with AmB and natural products, e.g. plumbagin, pedalitin, and fulvic acid, usually produced an additive or weak synergistic effect in a narrow concentration range^30,31^. It was also reported that simpotentin, which is a glycolipid composed of a mannosyl group with two medium-chain fatty acids, produced by *Simplicillium minatense* potentiates AmB activity against *C*. *albicans* determined with the *in vitro* microdilution method^32^. In our research team, the search for molecules showing synergistic interactions with AmB was focused on a group of synthetic 1,3,4-thiadiazole derivatives, which are reported as promising antimicrobial agents^33–35^. The range and mechanism of the antifungal activity of compounds belonging to this group is poorly understood. Among the studied derivatives of 5-substituted 4-(1,3,4-thiadiazol-2-yl) benzene-1,3-diols, only a compound called 4-(5-methyl-1,3,4-thiadiazole-2-yl) benzene-1,3-diol (abbreviated as C1) showed strong synergistic interaction with AmB and low toxicity towards human cells. This compound has a relatively simple structure (Fig. 1), in which the heterocyclic ring of 1,3,4-thiadiazole is substituted by the benzene-1,3-diol in the 2-position and by the methyl group in the 5-position and is used as a scaffold for the synthesis of more biologically active derivatives possessing antifungal, antitumor, and neuroprotective potential^35–39^. As shown in model studies with 1,2-dipalmitoyl-sn-glycero-3-phosphatidylcholine (DPPC) liposomes, the C1 compound molecules were located close to the polar lipid heads on the lipid-water interface and exhibited no interactions with the core part of the lipid membrane^40^. This property of the compound may be associated with its low cytotoxicity to animal cells. The minimal inhibitory concentration (MIC) for the C1 compound was studied against several *Candida* strains and had a mean value of 67.2 (±38,4) µg/mL^41^. Such doses are difficult to achieve *in vivo*, which limits the practical application of this compounds in monotherapy of systemic fungal infections. To date, the antifungal activity of polyene antibiotics in combination with 1,3,4-thiadiazole derivatives has not been investigated. The aim of the present study was to investigate the type of *in vitro* antifungal interactions of the C1 compound with AmB against clinical isolates of fungal pathogens with different susceptibility to antifungals.

## Material and Methods

### Antifungals

Amphotericin B powder from *Streptomyces sp*. was obtained from Sigma-Aldrich (cat. No A4888). The purity of the antibiotic powder (HPLC) was about 80%, which was taken under consideration when calculating concentrations. AmB was dissolved in alkalized water (adjusted to pH 12.6 with 1 N NaOH) at the concentration of 1 mg/mL and immediately diluted with the culture medium to obtain the stock solution with the concentration of 10 µg/mL. The AmB stock solution was prepared directly before each experiment to avoid loss of its activity. Synthesis of 4-(5-methyl-1,3,4-thiadiazole-2-yl) benzene-1,3-diol (C1) was carried out as described by Matysiak *et al*. 2006. The stock solution was obtained by dissolving 1 mg of C1 in 100 µL of DMSO and was further diluted with the culture medium to the final concentration of 1 mg/mL. The chemical structure of AmB and the C1 compound are presented in Fig. 1.

### Fungal strains

The reference strains of *Candida albicans* NCPF 3153, *Candida parapsilosis* ATCC 22019, *Trichophyton rubrum* ATCC 28188, *Rhodotorula mucilaginosa* ATCC 22273, and *Aspergillus niger* ATCC 16888, as well as the clinical fungal isolates of *Candida albicans* isolate 102 (itraconazole resistant), *Candida krusei* isolate 93 (fluconazole and itraconazole resistant), *Candida dubliniensis* isolate 176 (itraconazole and flucytosine resistant), *Candida glabrata* isolate 124 (moderately fluconazole and itraconazole sensitive), and *Candida tropicalis* isolate 175 (fluconazole, itraconazole, and voriconazole resistant) were used. The isolates were obtained from the intensive care unit of the Independent Teaching Hospital No 4 in Lublin, Poland. The collection and transport of the biological material were carried out according to the hospital procedure (ZSZ procedure no: PM SE/010, 3rd edition, “Transport and collection of diagnostic materials for clinical trials”). Collected swabs were selectively cultured on Sabouraud 2 Chloramphenicol agar medium (BioMerieux). Identification of the recovered colonies to the species level was made using *Candida* Chromogenic LAB-AGAR (BioMaxima, BioCORP) and ID 32 C (BioMerieux) tests, based on the metabolic profiles of individual species. The susceptibility of the pathogenic fungal isolates to the commonly applied antifungals was studied with the ATB Fungus 3 strips reading method (BioMerieux), and interpretation of the antibiogram followed the EUCAST 8.8 (2018) recommendation. The identification of fungal isolates and their susceptibility tests were performed in the Laboratory for Microbiological Diagnostics in the Chair and Department of Pharmaceutical Microbiology of Medical University in Lublin (number 2683 on the list of the National Chamber of Laboratory Diagnosticians in Poland). After identification and determination of their susceptibility, the reference strains and the clinical isolates were stored in a cryoprotective medium of VIABANK (BioMaxima) at -70 °C. Before each experiment, in order to obtain fungal cultures in the logarithmic growth phase, the strains were inoculated into a liquid YPD medium (1% yeast extract, 2% bactopepton, 2% glucose) in phosphate buffer pH 7.0 and precultured at 35 °C for 24 h with shaking.

### Determination of the type of interaction between AmB and C1

The antifungal activity of AmB and C1 separately was determined using the broth microdilution method by identification of minimal inhibitory concentrations (MIC). Standard methodological guidelines recommended by the Clinical and Laboratory Standards Institute (CLSI): document M27-A3^42^ and M38-A2^43^ for testing the susceptibility of yeasts and moulds, respectively, were applied. The fungal inoculum with a final density of 0.5 × 10^3^–2.5 × 10^3^ cells/mL for yeast and 0.4 × 10^4^–5 × 10^4^ for mould spores was prepared in a culture medium. The RPMI 1640 medium without phenol red and sodium bicarbonate (Sigma-Aldrich, R8755) was used. The medium was buffered to pH 7.0 with 0.165 mol/L 3-(*N*-morpholino) propanesulfonic acid (MOPS) and supplemented with 2% of glucose to optimize the conditions for fungal growth. The same medium was used for fungal cultures in all experiments. The stock solutions of AmB and C1 were used to obtain the final dilutions in the broth medium on 96-well microtitre plates. The final concentration of DMSO did not exceed 0.5% of the culture medium, and a control of fungal growth was performed in the medium supplemented with the solvent. After inoculation, the microtitre plates were incubated for 48 h at a temperature of 35 °C or 27 °C, for yeasts and moulds, respectively. The optical density was determined spectrophotometrically using an E-max Reader microplate reader at a wavelength of 600 nm. 100% inhibitory concentrations were taken as the MIC values.

The type of interaction of AmB and C1 was analyzed with the checkerboard microdilution method, in 96-well microtitre plates, as described in many publications^24,44,45^. This method gives a matrix of the concentrations of two compounds in different combinations. The final concentration range was 0.015–4 µg/mL for AmB and 0.5–128 µg/mL for C1. To prepare the combined concentrations of the two compounds, 200 µL aliquots of the 4-fold concentrated AmB and C1 solutions were pipetted into wells in the first horizontal (AmB) and vertical (C1) row. The AmB solution was serially diluted vertically, and next it was cross-diluted horizontally with the C1 solution. Separate rows of wells were prepared with AmB and C1 diluted separately, with the control medium, and with the solvent-containing medium. The inoculation and the culture conditions were carried out according to the above-described procedure. A combined concentration of the two compounds that caused 100% fungal growth inhibition after 48 h of culture was assumed as the MIC value. The interaction between the two compounds was interpreted based on the calculated coefficient of the sum of fractional inhibitory concentrations (∑FIC), in accordance with the recommendations of the American Society for Microbiology^46^. The coefficient was calculated from the formula: ∑FIC = FIC_AmB_ + FIC_C1_, where: FIC_AmB_ = MIC_AmB_ in the presence of C1/MIC_AmB_ separately and FIC_C1_ = MIC_C1_ in the presence of AmB/MIC_C1_ separately. The following interaction criteria were applied: ∑FIC ≤ 0.5 - synergy, ∑FIC > 0.5 do ≤ 1 - additivity, ∑FIC > 1 do < 2 - no interaction, ∑FIC ≥ 2 - antagonism.

To determine whether the earlier exposure of fungal cells to C1 induces their resistance to AmB, an experiment was conducted, in which the susceptibility of the strains not exposed earlier to C1 (group 1) and exposed to C1 at a concentration of ½ MIC24 for 24 h (group 2) was compared. In this experiment, the procedure of the MIC and the ΣFIC coefficient determination was carried out as described above.

### Morphological observations of fungal cells

For morphological observations, the *C*. *albicans* and *C*. *parapsilosis* reference strains were cultured in the presence of AmB and C1 separately and in combination, according to the checkerboard microdilution method, in the medium supplemented with 20% bovine serum to induce formation of hyphae. The cell pellet collected from the bottom of the wells with concentrations of the antifungals below the MIC values was used to prepare microscope slides. To visualize the general fungal cell morphology, chitin staining was performed using a calcofluor white solution (Sigma-Aldrich, 18909). The fungal cells were placed in a solution containing 1 g/l of calcofluor white and 0.5 g/l of Evans blue for 5 min; next, they were centrifuged and washed in PBS buffer, pH 7.4. A drop of stained cell suspension was placed on a microscopic slide, covered with a cover glass, and observed under Nikon Labophot 2 fluorescence microscope using a 380–420 nm band-pass blue excitation filter block (V-2A, Nikon). Calcofluor white interacts with chitin in fungal cell walls and gives bright blue fluorescence. A series of microphotographs was taken from each slide using a Canon Power Shot A 640 digital camera.

To visualize the intracellular acidic compartments in fungal cells, staining with a solution of acridine orange hydrochloride hydrate (Sigma-Aldrich, 318337) was used. Fungal cells treated with AmB and C1 separately or in combination at the concentration range below the MIC values were collected from the culture plate wells and stained with a water solution of 1 µg/mL of acridine orange. The acridine orange is a fluorochrome that penetrates cell membranes and fluoresces orange-red in low pH-compartments, e.g. vacuoles and lysosomes^47,48^. Freshly prepared microscope slides were examined under the fluorescence microscope equipped with a 450–490 nm band-pass blue excitation filter block (B-2A, Nikon).

### Testing the fungal cell membrane integrity

To study the influence of the tested antifungals on the cell membrane integrity, the reference strains of *C*. *albicans* and *C*. *parpsilosis* at the initial inoculum density of 1 × 10^4^–1 × 10^5^ were grown in the RPMI 1640 medium with addition of 1–32 µg/mL of C1 for 24 h or treated with AmB at the concentration range of 0.03–2 µg/mL for 4 h. To obtain cells treated with the combination of both antifungals, cultures grown in the presence of C1 for 24 h were subsequently exposed to AmB for 4 h. The combination treatment was performed with the checkerboard microdilution method. At the appointed time, the cell pellet was aspired from the bottom of the wells and stained with the reagent kit LIVE/DEAD FungaLight Yeast Viability (Life Technologies, L34952). This reagent contains a mixture of two nucleic acid stains: SYT0 9 giving green fluorescence and propidium iodide (PI) giving red fluorescence. The SYT0 9 stain penetrates both cells with intact membranes and cells with damaged membranes, while the PI stain accumulates only in cells with damaged membranes. Both dyes are present in cells with damaged cell membrane, but only red fluorescence is visible, because the SYT0 9 green fluorescence is reduced by fluorescence resonance energy transfer (FRET). The excitation/emission maximum is 480/500 nm for SYT0 9 and 490/635 nm for PI; therefore, for the observations of the slides, the microscope was equipped with a 450–490 nm band-pass blue excitation filter block (B-2A, Nikon), a 500 nm dichromatic mirror, and a 515 nm long-pass barrier filter.

### Testing the level of oxidative stress in fungal cells

To test the level of oxidative stress, the fungal cultures were prepared according to the procedure described for membrane integrity testing. The level of oxidative stress was tested using 2′,7′-dichlorofluorescin diacetate (H_2_DCF-DA), i.e. a cell permeable nonfluorescent probe, which is de-esterified intracellularly to its polar but not fluorescent form (H_2_DCF) trapped inside the cell. Upon oxidation by intracellular ROS, the molecule turns into a highly fluorescent form (DCF). H_2_DCF-DA is regarded as an indicator of the degree of general oxidative stress in cells^49^. At the appointed time, cells from the wells were aspired, centrifuged, and washed in PBS buffer, pH 7.4. The reagent 2′,7′-dichlorofluorescin diacetate (Sigma-Aldrich D6883) was dissolved immediately before use in anhydrous DMSO to obtain a 10 mM stock solution. 1 µL of the reconstituted dye was diluted in 1 mL of PBS to give a final concentration of 10 µM and vortexed to disperse the dye evenly. The fungal cells were stained with the H_2_DCF-DA solution in the dark for 10 min and washed in PBS. The stained cells were placed on microscope slides and mounted in a drop of VECTASHIELD Antifade Mounting Medium. The slides were captured immediately using a Nikon Labophot 2 fluorescence microscope equipped with a 450–490 nm band-pass blue excitation filter block (B-2A, Nikon). Additionally, the induction of intracellular oxidative stress was determined using dihydrorhodamine 123 (DHR 123) (Sigma-Aldrich, cat. no D1054) and MitoSOXRed mitochondrial superoxide indicator (ThermoFisher, cat. no M36008) according to a procedure described earlier^50^.

### Evaluation of cytotoxic activity

The cytotoxicity of C1 administered separately and in combination with AmB against normal human dermal fibroblasts (NHDF, Lonza, CC-2511, Bazel, Switzerland) was determined in *in vitro* culture. The cells were stored in liquid nitrogen in a tissue bank, thawed at 37 °C, and pre-cultured in a Dulbecco’s modified Eagle medium nutrient mixture F-12 HAM (Sigma, D8062) supplemented with 10% thermal inactivated fetal bovine serum in bottles for adherent cultures (Nunclon) placed in an incubator with humidified atmosphere saturated with 5% CO_2_ at 37 °C. The NHDF cells were cultured to 80%-confluence, harvested using a 0.25% trypsin/EDTA solution, and seeded into 96-well plates at a density of 4 × 10^3^ viable cells per well. After 24 h of culture, the medium was poured out and 200 µL of fresh medium heated to 37 °C were added to each well. Next, AmB and C1 stock solutions were added to obtain the desired final concentrations. Due to the limited number of rows on the 96-well plate, two experiments were carried out with various concentrations of AmB. In the first experiment, AmB at the final concentrations of 1 and 5 µg/mL and C1 at the final concentrations of 10, 20 and 40 µg/mL were added separately and in combination. In the second experiment, higher concentrations of AmB (10 and 20 µg/mL) were added to the 10, 20, and 40 µg/mL C1 solutions. 72 h after administration of AmB and C1, cell viability was determined using the *In Vitro* Toxicology Assay Kit, MTT based (Sigma, TOX 1MTT assay), according to the manufacturer’s instructions. The amount of produced formazan was determined spectrophotometrically using an E-max Reader microplate reader at a wavelength of 570 nm. The results are shown as the percent of the control.

### Statistical analysis

All determinations were made in three independent experiments and at least 6 replicates. The mode values were taken in the MIC determination using the checkerboard microdilution method. To compare the differences between the means in the cytotoxicity studies, a one-way analysis of variance (ANOVA) and post-hoc Tukey tests were performed in the StatSoft Statistica 12.5 software.

## Results

### Antifungal interactions of AmB with C1 in combination treatment

**Figure 2:**
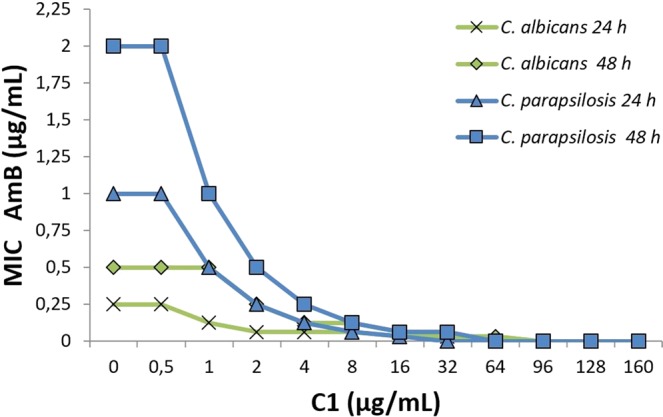
MIC of AmB in the presence of different concentrations of the C1 compound determined for the reference strains of *C*. *albicans* NCPF 3153 and *C*. *parapsilosis* ATCC 22019 using the checkerboard microdilution method after 24 and 48 h of culture.

The studies of the type of antifungal interactions of AmB and the C1 compound were performed with the checkerboard microdilution method using reference strains of *C*. *albicans*, *C*. *parapsilosis*, *T*. *rubrum*, *R*. *muciaginosa*, and *A*. *niger* as well as azole-resistant clinical isolates of *C albicans*, *C*. *krusei*, *C*. *dubliniensis*, *C*. *glabrata*, and *C*. *tropicalis*. The tested strains were characterized by varying susceptibility to AmB (MIC values ranging from 0.25 to 0.5 μg/mL) and to the C1 compound (MIC values ranging from 8 to 96 μg/mL) (Table 1). To obtain the most reliable results, the readings were performed after 48 h of culture, and 100% inhibition of fungal growth was taken as the MIC value. With the use of the checkerboard microdilution method, in which the concentrations of the two compound combinations were studied, it was possible to determine the sum of fractional inhibitory concentrations (ΣFIC), which indicate the type of the interaction between the two compounds. Synergistic (∑FIC ≤ 0.5) or additive (∑FIC > 0.5 do ≤ 1) interactions of AmB and C1 were found for all tested strains in a concentration range depending on the sensitivity of a given pathogen. For strains with higher sensitivity to C1 separately (with MIC values of 8–64 μg/mL), the synergistic interactions with AmB were found in the range of C1 concentrations from 2 to 32 μg/mL. For strains with lower susceptibility to C1 (MIC value of 96 µg/mL), such as *C*. *glabrata*, *C*. *tropicalis*, and *C*. *krusei*, synergistic interactions with AmB were observed in the C1 concentration range of 8–32 μg/mL, whereas the lower doses of C1 showed additivity or lack of interactions (∑FIC > 1 do < 2) (Table 1). No antagonistic interactions (∑FIC ≥ 2) were found in any of the tested strains at any concentration. It was observed that, with the increasing dose of C1, the concentration of AmB necessary for 100% inhibition of fungal growth decreased, and the dynamic of this reduction depended on the individual sensitivity of the strains. Analysis of Tables 1 and 2 suggests that the 16 μg/mL-C1 dose had the synergistic effect with AmB in most of the strains and reduced the AmB concentration necessary for 100% inhibition of fungal growth by 4–32 times. At the C1 doses of 2–8 μg/mL, the synergistic interactions producing a several-dozen-fold decrease in the AmB MIC value were observed for most of the strains (Tables 1 and 2). It is particularly important to observe that that 4–32 fold reduction in the AmB dose in the C1 concentration ranging between 2 and 32 μg/mL was achieved in the case of strains with reduced sensitivity to AmB, such as *C*. *parapsilosis* (AmB MIC equal to 2 μg/mL). The dependence of the AmB MIC on the dose of C1 for the *C*. *albicans* and *C*. *parapsilosis* reference strains is shown graphically in Fig. 2.

**Table 1:** Interactions of 4-(5-methyl-1,3,4-thiadiazol-2-yl) benzene-1,3-diol (denoted by C1) with amphotericin B (AmB) in *in vitro* combating pathogenic fungal isolates and reference strains obtained after 48-h culture with the checkerboard microdilution method; 100% inhibition of growth was taken as the MIC value.

Strain	Separate treatment		Combinatory treatment											
			2 µg/mL C1		4 µg/mL C1		8 µg/mL C1		16 µg/mL C1		32 µg/mL C1		64 µg/mL C1	
	MIC C1 [µg/mL]	MIC AmB [µg/mL]	MIC AmB [µg/mL]	∑FIC	MIC AmB [µg/mL]	∑FIC	MIC AmB [µg/mL]	∑FIC	MIC AmB [µg/mL]	∑FIC	MIC AmB [µg/mL]	∑FIC	MIC AmB [µg/mL]	∑FIC
*Candida albicans* isolate 102	32	0.5	0.25	0.56*	0.125	0.38**	0.0625	0.37**	0.0312	0.56*	—	—	—	—
*Candida krusei* isolate 93	64	0.5	0.25	0.53*	0.125	0.31**	0.0625	0.24**	0.0312	0.31**	0.0156	0.52*	—	—
*Candida dubliniensis* isolate 176	8	0.5	0.0625	0.37**	0.0078	0.52*	—	—	—	—	—	—	—	—
*Candida glabrata* isolate 124	96	1.0	1.0	1.02	1.0	1.04	1.0	1.17	0.25	0.42**	0.0078	0.34**	0.0078	0.67*
*Candida krusei* isolate 103	96	1.0	1.0	1.02	0.5	0.54*	0.25	0.33**	0.25	0.42**	0.0625	0.39**	0.0078	0.67*
*Candida tropicalis* isolate 175	96	1.0	1.0	1.02	1.0	1.04	0.5	0.58*	0.25	0.42**	0.0625	0.39**	0.0078	0.67*
*Trichophyton rubrum* ATCC 28188	32	0.25	0.125	0.31**	0.125	0.37**	0.125	0.5**	0.0625	0.62*	—	—	—	—
*Rhodotorula mucilaginosa* ATCC 22273	32	1.0	0.5	0.56*	0.25	0.37**	0.125	0.37**	0.0312	0.53*	—	—	—	—
*Aspergillus niger* ATCC 16888	64	1.0	0.125	0.16**	0.0625	0.12**	0.0312	0.16**	0.0312	0.28**	0.0312	0.53*	—	—
*Candida albicans* NCPF 3153	96	0.5	0.25	0.52*	0.125	0.29**	0.125	0.33**	0.0625	0.29**	0.0312	0.40**	0.0312	0.73*
*Candida parapsilosis* ATCC 22019	64	2.0	0.5	0.28**	0.25	0.19**	0.125	0.19**	0.0625	0.28**	0.0625	0.53*	—	—

The MIC value of AmB [µg/mL] in the presence of the C1 in a concentration range of 2–64 g/mL and ∑FIC (sum of fractional inhibitory concentrations) are presented. The interpretation of the interactions is shown as follows: **synergy (∑FIC ≤ 05), *additivity (∑FIC > 0.5 to ≤1), lack of a label means a non-differentiating effect (∑FIC > 1 to < 2), no antagonistic interactions (∑FIC ≥ 2) were observed in any combination of the concentrations. ∑FIC = FIC_AmB_ + FIC_C1_, where: FIC_AmB_ = MIC_AmB_ in the presence of C1**/**MIC_AmB alone_; FIC_C1_ = MIC_C1_ in the presence of AmB/MIC_C1 alone_.

**Table 2:** Interactions of 4-(5-methyl-1,3,4-thiadiazol-2-yl) benzene-1,3-diol (denoted by C1) with amphotericin B (AmB) in *in vitro* combating pathogenic fungal isolates and reference strains obtained after 48-h culture with the checkerboard microdilution method; 100% inhibition of growth was taken as the MIC value. The reduction factor shows how many times the AmB MIC is reduced at the different C1 concentrations in the combinatory treatment.

Strain	Separate treatment		Combinatory treatment					
			Reduction factor of AmB MIC at following C1 concentrations					
	MIC C1 [µg/mL]	MIC AmB [µg/mL]	C1 2 µg/mL	C1 4 µg/mL	C1 8 µg/mL	C1 16 µg/mL	C1 32 µg/mL	C1 64 µg/mL
*C*. *albicans* isolate 102	32	0.5	2	4	8	16	—	—
*C*. *krusei* isolate 93	64	0.5	2	4	8	16	32	—
*C*. *dubliniensis* isolate 176	8	0.5	8.3	64	—	—	—	—
*C*. *glabrata* isolate 124	96	1	1	1	1	4	128.2	128.2
*C*. *krusei* isolate 103	96	1	1	2	4	4	16	128.2
*C*. *tropicalis* isolate 175	96	1	1	1	2	4	16	128.2
*T*. *rubrum* ATCC 28188	32	0.25	2	2	2	4	—	—
*R*. *mucilaginosa* ATCC 22273	32	1	2	4	8	32	—	—
*A*. *niger* ATCC 16888	64	1	8	16.6	32	32	32	—
*C*. *albicans* NCPF 3153	96	0.5	2	4	4	8	16	16
*C*. *parapsilosis* ATCC 22019	64	2	4	8	16	32	32	—

**Figure 3:**
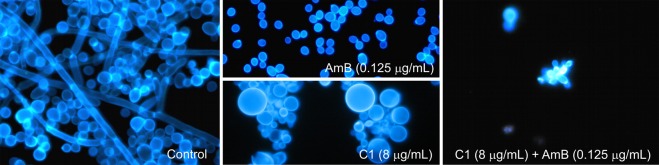
Morphological images of *C*. *albicans* cells from the control culture and cultures treated with AmB or C1 separately or in combination of AmB and C1 for 24 h. Fluorescence microscope; staining with calcofluor white detecting chitin in the cell walls; magnification 600×.

**Figure 4:**
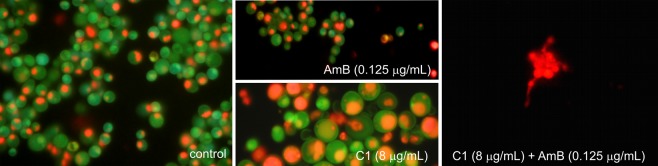
Morphological images of *C*. *albicans* cells from the control culture and cultures treated with AmB or C1 separately or in combination of AmB and C1 for 24 h. Fluorescence microscope; staining with acridine orange, acidic compartments fluoresce in orange; magnification 600×.

The microscopic observations of fungal cultures treated with AmB and C1 separately or in combination confirmed the results obtained with the checkerboard microdilution method. Representative microphotographs of the control *C*. *albicans* cells and the cells treated with AmB (at the concentration of 0.125 μg/mL) or with C1 (at the concentration of 8 μg/mL) separately and in combination are shown in Figs 3 and 4. The morphological disturbances in the fungal cells induced by the tested antifungals were observed after staining with the calcofluor white dye, i.e. a fluorochrome staining the chitin in fungal cell walls (Fig. 3). It was observed in an experiment performed in a culture medium inducing formation of hyphae that both AmB and C1 used separately or in combination inhibited the formation of hyphae. This observation is important, as hyphae and pseudohyphae are regarded as the main invasive forms of this pathogen. In addition, under the influence of the low doses of AmB, a decrease in the cell size was observed, while numerous morphological disturbances were induced by C1. Upon the C1 treatment, formation of giant cells next to dwarfed cells and intense flocculation were observed. The treatment with the combination of AmB and C1 at the concentrations mentioned above resulted in the death of most of cells. As shown in Fig. 3, a few remaining cells displayed severe disturbed morphology upon the combined AmB and C1 treatment.

Similar observations were made in cells stained with acridine orange, i.e. a fluorochrome that visualizes cellular regions with low pH in orange-red color. In the control *C*. *albicans* cells, there were small fluorescing vacuoles with a regular shape (Fig. 4). Quite a similar image was obtained in cells treated with the low doses of AmB. In contrast, a significant increase in intracellular regions with low pH was observed in the C1-treated cells. In some cells, the occurrence of numerous vacuoles with irregular shapes or orange-red fluorescence of the entire cytoplasm was visualized. These images indicate serious disturbances of cellular organelles under the influence of C1. The use of the combination of both antifungals resulted in a significant reduction in the number of cells, and severe morphological disturbances. A few remaining cell aggregates were entirely intensely red, which indicated the loss of integrity in these cells. The microscopic observations confirmed the synergistic antifungal effect of AmB and C1 and pointed to different mechanisms of action of both compounds.

**Figure 5:**
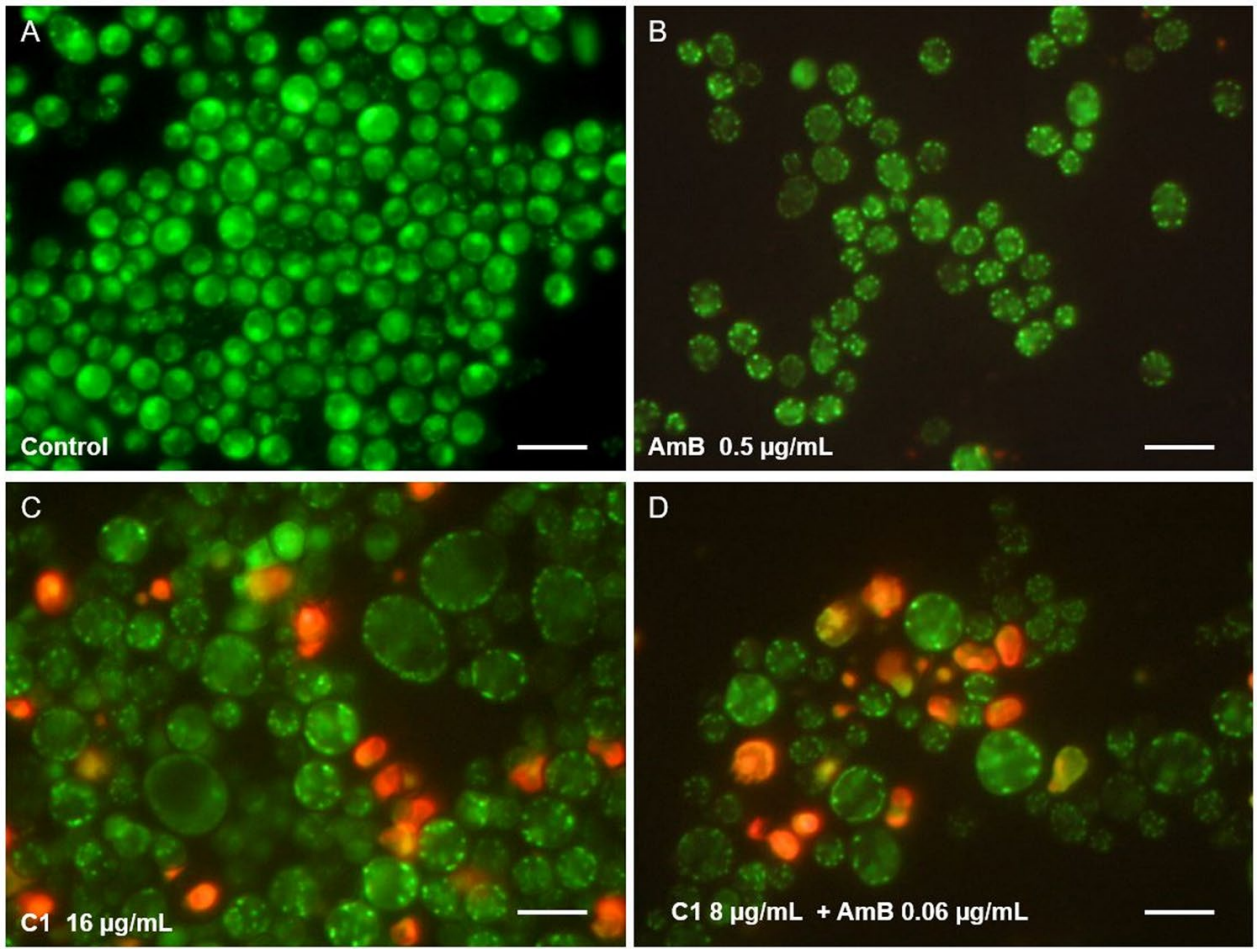
Detection of the cell membrane damage in *C*. *albicans* cells from the control culture and cultures treated with AmB or C1 separately or in combination of AmB and C1. The cultures were treated with C1 for 24 h and with AmB for 4 h. Fluorescence microscope; staining with LIVE/DEAD FungaLight Yeast Viability Kit, containing mixture of two dyes: SYT0 9, the membrane permeating green-fluorescent stain, and propidium iodide red-fluorescent stain penetrating only cells with damaged plasma membrane. Scale bar 10 µm.

In the next experiment, the effect of a combination treatment with AmB and C1 on the integrity of the fungal cell membrane was investigated. For this purpose, staining with a mixture of two fluorochromes: propidium iodide (PI) and SYT0 9 was performed. Propidium iodide, i.e. a red-fluorescent stain, penetrates only cells with damaged plasma membrane and the SYT0 9, which is a membrane permeating green-fluorescent stain, visualizes cells with intact plasma membrane. In this experiment, the fungal cultures were treated with C1 for 24 h and with AmB for 4 h, due to the different mechanisms of action of both compounds (see the discussion section). The representative microphotographs from staining in the *C*. *albicans* reference strain are shown in Fig. 5. The images indicated that AmB does not cause direct loss of the fungal cell membrane integrity, and the accumulation of PI appeared only at the last stage of cell death. Also upon the C1 treatment, no loss of cell membrane integrity was observed, even in the giant cells. The red color was only observed in the group of dwarfed cells at the advanced stage of the cell death. Quite similar images were observed in the cultures treated with combination of both antifungals (Fig. 5).

**Figure 6:**
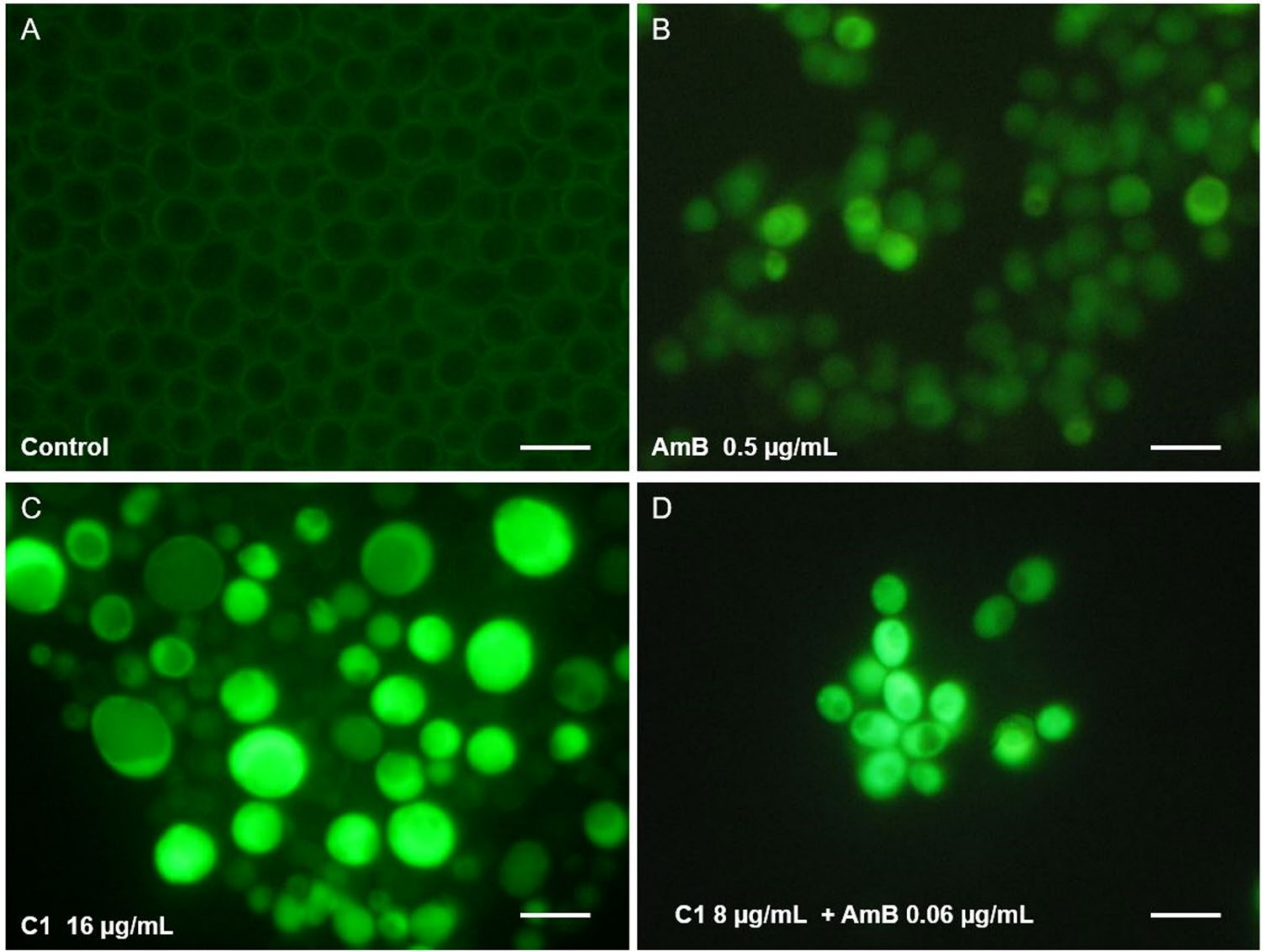
Detection of oxygen-reactive species in *C*. *albicans* cells from the control culture and cultures treated with AmB or C1 separately or in combination of AmB and C1. The cultures were treated with C1 for 24 h and with AmB for 4 h. Fluorescence microscope; staining with 2′,7′-Dichlorofluorescin diacetate. Scale bar 10 µm.

Interesting results were obtained in the staining with 2′,7′-dichlorofluorescin diacetate (H_2_DCF-DA), i.e. a probe that facilitates detection of general oxidative stress in cells. Cell membranes are freely permeable to esterified forms of H_2_DCF-DA. Intracellularly, the H_2_DCF-DA molecules are hydrolyzed by esterases into polar but not fluorescent forms (H_2_DCF), which become trapped inside the cell. Upon oxidation by reactive oxygen species (ROS), the molecule turns to highly fluorescent DCF. This probe allows sensitive and rapid quantitation of oxygen-reactive species in response to oxidative stress. Representative images obtained in this staining are shown in Fig. 6. Staining of the control *Candida* cells showed no fluorescence in the cytoplasm, which means that the polar form of H_2_DCF had not been oxidized inside the cells to the fluorescent form. Otherwise, dim fluorescence was observed in the cytoplasm of most of the AmB-treated cells. Intense fluorescence was also visible in some of cells upon the 4-hour AmB treatment. In turn, much stronger fluorescence was observed in the C1-treated cells. Particularly intense oxidative stress was noticed in the giant cells, while less intense fluorescence was observed in the standard sized cells and in the dwarfed cells. Upon the combination treatment with C1 and AmB, an increase in fluorescence intensity was observed in all cells. In order to confirm the results of oxidative stress induction, additional studies were conducted using other fluorescent ROS indicators. Dihydrorhodamine 123 (DHR 123) was used for detection of intracellular ROS and MitoSOX Red was applied for detection of mitochondrial superoxide. DHR 123 is an uncharged and nonfluorescent molecule that can passively diffuse across membranes where it is oxidized to cationic rhodamine 123, which localizes in the mitochondria and exhibits green fluorescence. The MitoSOX Red reagent is live-cell permeant; it is targeted rapidly and selectively to the mitochondria, where it is oxidized by superoxide and exhibits red fluorescence. The results obtained with the last two ROS indicators confirmed the data obtained for H_2_DCF-DA (supplementary materials Figs 1 and 2). It can be concluded that both antifungals induce oxidative stress in fungal cells and their combination induces a high-intensity oxidative stress, which causes irreversible cell damage.

### Antifungal interactions of AmB with C1 in the cells pre-exposed to C1

Literature reports indicate that antifungal drugs from the azole group applied in maintenance therapy can induce the resistance of fungal pathogens to AmB. To determine whether the earlier exposure of fungal strains to C1 induces their resistance to AmB, an experiment was conducted, in which the susceptibility of strains that were either not exposed to C1 (group 1) or exposed to C1 at a concentration of ½ MIC_24_ for 24 h (group 2) was compared. The results showed that the pre-exposure to C1 did not change sensitivity or decreased tolerance to both C1 and AmB, depending on the strain (Table 3). In the case of *C*. *albicans*, the AmB and C1 MIC values remained unchanged for cells that had been previously treated with C1, in comparison to cells that had not been not previously exposed to this substance. In the case of *C*. *parapsilosis*, the pre-exposure to C1 caused a two-fold reduction in the MIC value for both AmB and C1. It can therefore be concluded that pre-exposure to C1 does not induce AmB resistance in *Candida* species. The type of interaction of AmB and C1 in cells pre-exposed to C1 was similar to that of control cells (Table 4).

**Table 3:** Comparison of the susceptibility of the control and C1 pre-exposed (½ MIC for 24 h) *C*. *albicans* and *C*. *parapsilosis* reference strains to AmB and C1.

Strain	Control cells				Cells pre-treated with C1			
	MIC C1 [µg/mL]		MIC AmB [µg/mL]		MIC C1 [µg/mL]		MIC AmB [µg/mL]	
	24 h	48 h	24 h	48 h	24 h	48 h	24 h	48 h
*Candida albicans* NCPF 3153	64	96	0.25	0.5	64	96	0.25	0.5
*Candida parapsilosis* ATCC 22019	32	64	1	2	8	32	0.5	1

**Table 4:** Interactions of 4-(5-methyl-1,3,4-thiadiazol-2-yl) benzene-1,3-diol (denoted by C1) with amphotericin B (AmB) in combating *C*. *albicans* and *C*. *parapsilosis* reference strains pre-treated with C1 at the concentration of ½ MIC_24_ (16 µg/mL for *C*. *parapsilosis* and 32 µg/mL for *C*. *albicans*) obtained after 48-h culture with the checkerboard microdilution method; 100% inhibition of growth was taken as the MIC value. The MIC value of AmB [µg/mL] in the presence of C1 in a concentration range of 1–32 g/mL and ∑FIC (sum of fractional inhibitory concentrations) are presented. The interpretation of the interactions is shown as follows: **synergy (∑FIC ≤ 0.5), *additivity (∑FIC > 0.5 to ≤1), lack of a label means a non-differentiating effect (∑FIC > 1 to <2), no antagonistic interactions (∑FIC ≥ 2) were observed in any combination of the concentrations. ∑FIC = FIC_AmB_ + FIC_C1_, where: FIC_AmB_ = MIC_AmB_ in the presence of C1/MIC_AmB alone_; FIC_C1_ = MIC_C1_ in the presence of AmB/MIC_C1 alone_.

Strain	Separate treatment of cells pre-exposed to C1		Combinatory treatment of cells pre-exposed to C1											
			1 µg/mL C1		2 µg/mL C1		4 µg/mL C1		8 µg/mL C1		16 µg/mL C1		32 µg/mL C1	
	MIC C1 [µg/mL]	MIC AmB [µg/mL]	MIC AmB [µg/mL]	∑FIC	MIC AmB [µg/mL]	∑FIC	MIC AmB [µg/mL]	∑FIC	MIC AmB [µg/mL]	∑FIC	MIC AmB [µg/mL]	∑FIC	MIC AmB [µg/mL]	∑FIC
*Candida albicans* NCPF 3153 24 h-treatment	64	0.25	0.25	—	0.125	0.531*	0.125	0.563*	0.0625	0.375**	0.03125	0.375**	0.03125	0.625*
*Candida albicans* NCPF 3153 84 h-treatment	96	0.5	0.25	0.510*	0.25	0.521*	0.25	0.542*	0.125	0.333**	0.0625	0.292**	0.0625	0.458**
*Candida parapsilosis* ATCC 22019 24 h-treatment	8	0.5	0.125	0.375**	0.0625	0.375**	0.0625	0.625*	—	—	—	—	—	—
*Candida parapsilosis* ATCC 22019 48 h-treatment	32	1.0	0.25	0.281**	0.25	0.312**	0.125	0.250**	0.0625	0.312**	0.03125	0.531*	—	—

No antagonistic interactions or non-differentiating effects were observed in any of the concentration combinations. These results clearly show that the C1 compound does not induce the resistance of *Candida* cells to AmB and does not change the type of interaction between these two antifungals.

### Evaluation of cytotoxic activity of combination of AmB and C1

**Figure 7:**
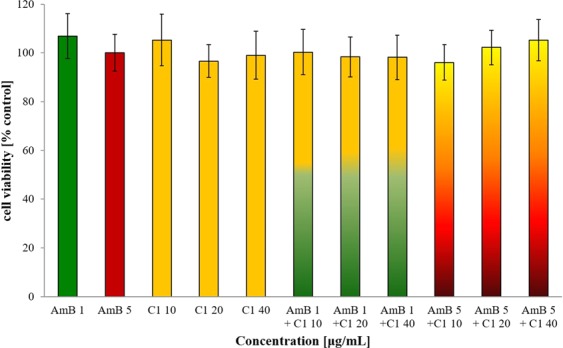
Viability of human skin fibroblasts (NHDF) after 96 h culture in the presence of the 1,3,4-thiadiazole derivative (C1) at the concentrations of 10, 20, and 40 µg/mL combined with 1 and 5 µg/mL of AmB, measured with the MTT assay; mean values with standard deviation are provided; the differences between treatment groups were not statistically significant (*p* > 0.05).

**Figure 8:**
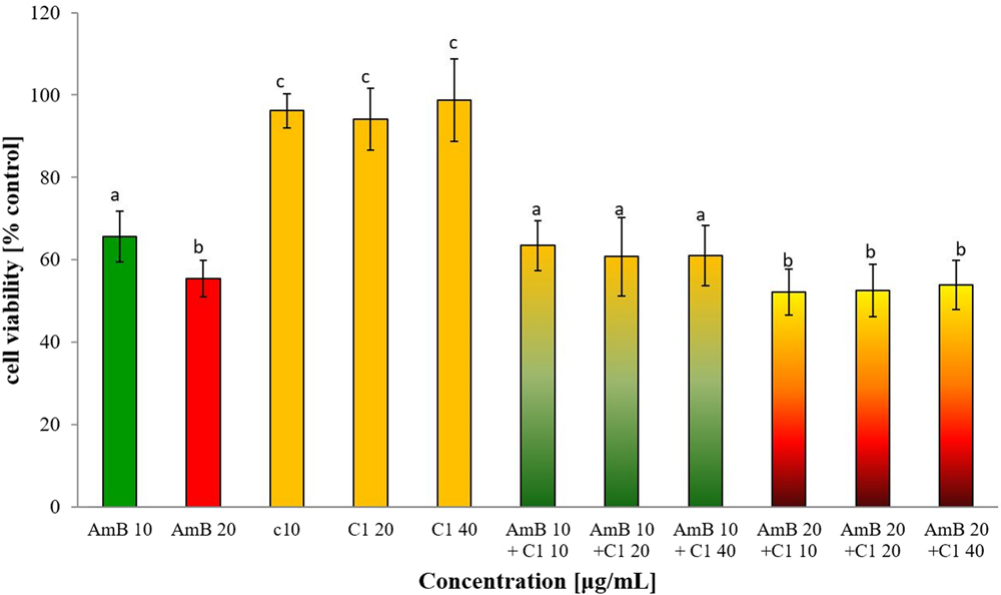
Viability of human skin fibroblasts (NHDF) after 96 h culture in the presence of the 1,3,4-thiadiazole derivative (C1) at the concentrations of 10, 20, and 40 µg/mL combined with 10 and 20 µg/mL of AmB, measured with the MTT assay; mean values with standard deviation are provided; the same letter means that the differences were not statistically significant (*p* > 0.05), different letters mean that the differences were statistically significant (*p* < 0.05).

The cytotoxicity of the C1 compound used separately against normal human dermal fibroblasts (NHDF) has been investigated in our previous work (in press). The IC_50_ value for this compound was determined at 512 μg/mL using the MTT-based spectrophotometric method for assessment of cell viability. The cytotoxic activity of AmB used separately against NHDF cells was previously determined as well, with the IC_50_ value of 17.46 ± 1.24 μg/mL for the Fungizone commercial preparation^50^. In the present study, it was determined whether the C1 compound administered in combination with AmB increases its cytotoxicity against NHDF cells *in vitro*. The results showed that the C1 compound administered together with AmB does not increase its cytotoxicity. The interaction of C1 at the concentrations of 10, 20, and 40 μg/mL with 1 and 5 μg/mL of AmB is shown in Fig. 7. Figure 8 shows the results of the experiment with the higher doses of AmB (10 and 20 μg/mL) used separately and in combination with 10, 20, and 40 μg/mL of the C1 compound. The results showed that the 10 and 20 μg/mL AmB doses reduced the NHDF cell viability to about 65% and 55% of the control level, respectively. The addition of C1 did not statistically significantly increase the cytotoxicity of AmB at the above-mentioned concentrations.

## Discussion

Searching for potential antifungal drugs showing synergistic interaction with AmB is a very important task, because most of the groups of antifungals used currently exhibit antagonism or lack of interactions with this antibiotic. AmB is a very potent antibiotic with very rare resistance among clinical isolates^1,4,51,52^. Given the severe side effects of AmB, immunocompromised patients receive drugs from the azole group as a maintaining treatment. AmB formulations are included only at aggravation of symptoms. However, as shown in many publications, the combined or sequential AmB treatment with ergosterol biosynthesis inhibitors (azoles) often not only fails to produce synergistic or additive effects but also produces antagonistic effects, since ergosterol is the target molecule for AmB. This is a serious problem in the treatment of mycoses, as ergosterol biosynthesis inhibitors can induce resistance of fungal pathogens to AmB^1,53–56^.

The results presented in this paper indicate that the C1 derivative of 1,3,4-thiadiazole shows strong synergistic interaction with AmB. The synergism allows for a dozen to several dozen times reduction in the AmB concentration necessary for 100% inhibition of the growth of pathogenic fungi *in vitro*. Synergistic interactions were noted for all the tested strains, including strains with reduced sensitivity to AmB and azole-resistant isolates. These observations give hope for the possibility of application of the AmB - C1 combinatory therapy in the treatment of fungal infections. Such a large reduction in the AmB dose would significantly restrict its side effects. As shown in *in vitro* cytotoxicity tests performed against the NHDF cell line, the combination treatment with the low doses of AmB and the high concentrations of C1 did not cause a decrease in cell viability. At the higher AmB doses (close IC_50_ values), the addition of C1 did not increase the cytotoxic effect of AmB. It was shown previously that the cytotoxicity of the C1 compound applied separately against NHDF was very low, with the IC_50_ value of 512 μg/mL (article in press). The low cytotoxicity of C1 is probably caused by the fact that this compound interacts with the hydrophilic region of the cell membrane and does not penetrate the cell cytoplasm^40^. Our previous studies suggested that the C1 compound interacts with the cell wall biogenesis in fungal cells at the cell surface. The disturbances in the cell wall integrity under the influence of C1 result in formation of giant cells, morphological disturbances, and premature cell death.

Depending on the assumed goal of the experiment, different methodological approaches were used while studying the antifungal activity of the C1 compound and its interaction with AmB. In the study aimed at determining the type of interactions, combinations of both compounds were administered simultaneously in accordance with the checkerboard microdilution method. This method allowed obtaining AmB MIC values at different C1 concentrations (Tables 1, 2, Fig. 2). In this experiment, a low-density inoculum was used (0.5 × 10^3^–2.5 × 10^3^ cells/mL, according to standard methodological guidelines recommended by CLSI) to determine the antibiotics doses that completely eliminate the growth of microorganisms. In such conditions, fungal cells proliferate only at sublethal doses of the antibiotics, and cells that survive and start divisions are in the recovery period, which makes it difficult to draw conclusions about the mechanism of action of the studied compounds. Representative morphological images of *C*. *albicans* cells treated for 24 hours with AmB and C1 administered simultaneously illustrate the synergistic action of both compounds (Figs 3 and 4). For microscopic imaging of the oxidative stress induction and the loss of cell membrane integrity, cultures with a higher initial inoculum density (10^5^ cells/mL) were prepared, whereby the doses of antibiotics at the MIC level were sublethal and it was possible to collect an appreciable amount of cells for preparation of slides. Additionally, the two compounds were not administered simultaneously, which resulted from our previous observations on the mechanism of the AmB action. AmB molecules interacting with ergosterol form transmembrane pores and cause loss of osmotic balance of fungal cells. It was shown that, after about 4 hours of AmB action, yeast cells underwent severe shrinkage and changes characteristic for apoptosis were observed in the cell cytoplasm and nucleus^57,58^. Induction of oxidative stress under the influence of AmB was detectable with the rhodamine 123 fluorescent probe just after 0.5 h and reached the maximum level after 2–4 hours. After a prolonged time of incubation (24 h) of *Candida* cells with the sublethal doses of AmB, the intensity of intracellular oxidative stress decreased and the cells entered the recovery phase without visible morphological changes^57^. The mechanism of the antifungal activity of the C1 compound, although not yet explained at the molecular level, is based on disturbances in the cell wall biogenesis (article in press). Such disturbances arise during the synthesis of the cell wall components, which is a slow process. Taking into consideration this information, an experiment was designed in which cells pretreated with sublethal doses of C1 for 24 h were then exposed to AmB for 4 h in order to observe induction of oxidative stress and loss of cell membrane integrity. The microscopic observations confirmed the synergistic antifungal effect of AmB and C1 and suggested diverse mechanisms of action of both antifungals. In the C1-treated cultures an increase in cell size and abnormalities of intracellular low pH - compartments were observed, suggesting necrotic or autophagic mechanisms of cell death. On the contrary, under the influence of AmB, the fungal cells decreased their size, became shrunken, and underwent apoptotic cell death. This is confirmed by the observation that, under the influence of AmB, the fungal cells had a long-lasting ability to exclude propidium iodide (PI), which was reported previously^57,58^. PI is a standard reagent used for assessing cell viability and distinguishing between necrotic and apoptotic cells. PI binds to double stranded DNA, but is excluded from cells with intact plasma membranes. The pores created by AmB together with ergosterol are permeable to K^+^ ions and small molecules, but have too small a diameter to let the PI molecules pass freely. Numerous observations indicate that AmB does not cause necrotic cell death associated with membrane damage, uncontrolled influx of water into the cell, swelling, and cell lysis. This phenomenon is probably caused by the presence of the cell wall, which balances the osmotic influx of water into the cells. Instead, the disturbance of the osmotic balance causes the death of fungal cells by apoptosis. Although the commonly accepted mechanism of the fungicidal activity of AmB is the binding of ergosterol and formation of transmembrane pores, many authors have reported that AmB-induced cell death is not a simple consequence of changes in the cell membrane permeability. Many studies have shown that the AmB effect results from sequestration of ergosterol, which performs vital functions in cell functioning^59^, and from induction of oxidative stress in cells^60–67^. While looking for an answer to the question about the mechanism of the synergistic interaction of AmB and C1, it should be noted that, according to the literature, cells with impaired cell wall integrity are hypersensitive to osmotic and oxidative stress^68,69^. Fungal cells under C1 treatment become morphologically altered due to incorrect cell wall biogenesis and hence may be more sensitive to the AmB action. Evidence for enhanced AmB activity in combination therapy with an echinocandin antifungal agent disrupting the cell wall biogenesis has been reported by other authors^29^. It was surprising that the induction of the oxidative stress under the influence of C1 was more intense than in the AmB treatment. To the best of our knowledge, the induction of the oxidative stress in fungal cells under a 1,3,4-thiadiazole derivative treatment is reported here for the first time. The induction of the oxidative stress by both interacting compounds may explain their synergistic interaction in fungal cell damage. Another aspect that may explain the synergistic interaction of AmB and C1 is that the disrupted integrity of the cell wall under the influence of C1 can facilitate penetration of AmB, which is a relatively large molecule, to the surface of the cell membrane and binding ergosterol, which is the target molecule. The discovery of the synergistic interaction between AmB and the C1 derivative from the group of 1,3,4-thiadiazoles is important from a practical point of view, because the application of such combination therapy in superficial and systemic mycoses is possible. This alternative therapy may turn out especially beneficial in the case of infections caused by azole- and echinocandin-resistant strains. The mechanism of the AmB-C1 interaction requires further studies. Elucidation of this mechanism can contribute to development of a more effective strategy in the designing of antifungal preparations.

## Supplementary information

Supplementary Dataset 1
