# Supplementary material for: Synergistic antifungal interactions of amphotericin B with 4-(5-methyl-1,3,4-thiadiazole-2-yl) benzene-1,3-diol
Source: Sci Rep. 2019 Sep 10;9:12945. doi: 10.1038/s41598-019-49425-1 (PMC6737028; doi:10.1038/s41598-019-49425-1)
Supplement: Supplementary file 1 — Supplementary Dataset 1 [file 41598_2019_49425_MOESM1_ESM.doc]

**Synergistic antifungal interactions of amphotericin B with 4-(5-methyl-1,3,4-thiadiazole-2-yl) benzene-1,3-diol**

Barbara Chudzik^1^*, Katarzyna Bonio^1^, Wojciech Dabrowski^2^**, Daniel Pietrzak^2^,
Andrzej Niewiadomy^3,4^, Alina Olender^5^, Katarzyna Malodobry^6^, Mariusz Gagoś^1^*


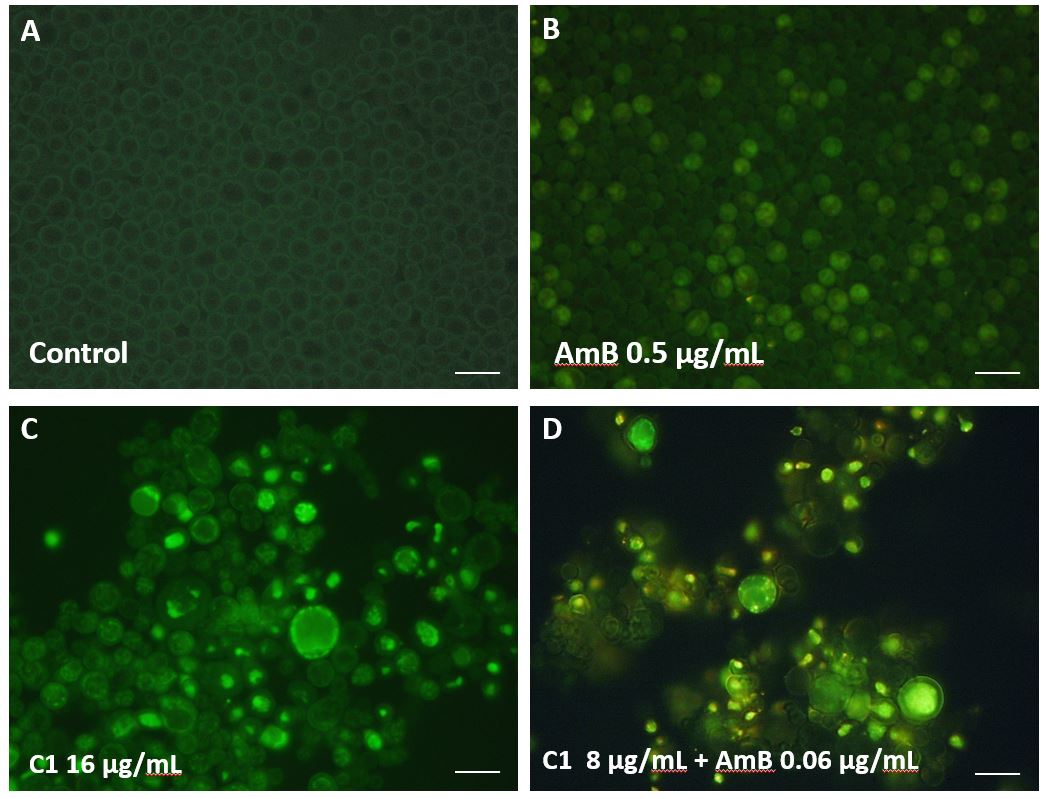


**Supplementary Fig. 1.** Detection of oxygen-reactive species in *C. albicans* cells from the control culture and cultures treated with AmB or C1 separately or in combination of AmB and C1. The cultures were treated with C1 for 24 h and with AmB for 4 h. Fluorescence microscope; staining with dihydrorhodamine 123 (DHR 123). Scale bar 10 µm.


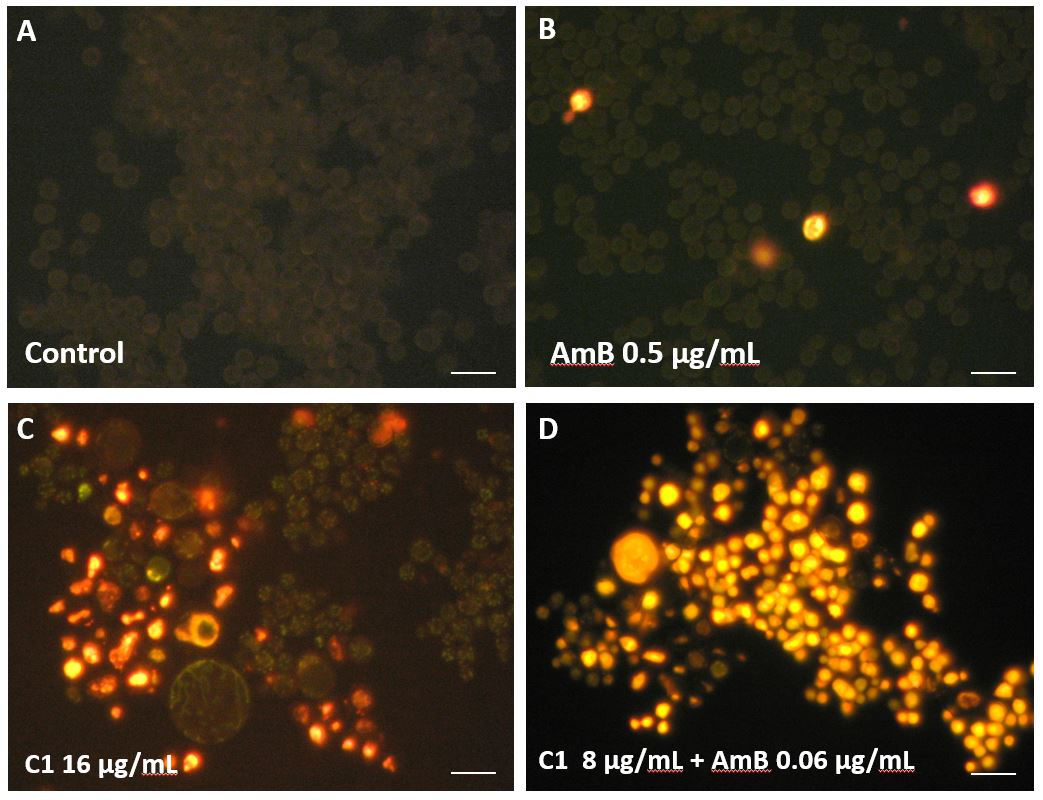


**Supplementary Fig. 2.** Detection of oxygen-reactive species in *C. albicans* cells from the control culture and cultures treated with AmB or C1 separately or in combination of AmB and C1. The cultures were treated with C1 for 24 h and with AmB for 4 h. Fluorescence microscope; staining with MitoSOX™ Red mitochondrial superoxide indicator (ThermoFisher, cat. no M36008). Scale bar 10 µm.
